# Impact of Green Tea Consumption on Postoperative Ileus in Colon Cancer Patients: A Pilot Randomized Controlled Trial

**DOI:** 10.7759/cureus.72157

**Published:** 2024-10-22

**Authors:** Jun Watanabe, Fuyumi Kobayashi, Makiko Tahara, Hiroyuki Kitabayashi, Mikio Shiozawa, Satoru Kondo, Masaru Koizumi

**Affiliations:** 1 Division of Gastroenterological, General and Transplant Surgery, Jichi Medical University, Shimotsuke, JPN; 2 Department of Surgery, Tochigi Medical Center Shimotsuga, Tochigi, JPN; 3 Division of Community and Family Medicine, Jichi Medical University, Shimotsuke, JPN

**Keywords:** colorectal neoplasm, : enhanced recovery after surgery (eras), green tea, length of hospital stay (los), postoperative ilues, randomized controlled trial (rct)

## Abstract

Introduction

The consumption of caffeine-rich green tea has been shown to promote recovery of postoperative gastrointestinal function after subtotal gastrectomy. However, the beneficial effects of green tea on colon cancer have not been clarified. This pilot study aimed to evaluate the impact of green tea intake on postoperative outcomes after colon cancer surgery.

Method

This study describes the findings of a single-center stratified randomized controlled trial. Colon cancer patients who underwent laparoscopic or restorative colon resection were randomly assigned to a postoperative green-tea-drinking or water-drinking group. The primary outcome was time to first defecation (hours). Secondary outcomes were the length of hospital stay, postoperative ileus, postoperative complications, mean daily fluid intake (mL), and the incidence of all adverse events. The protocol was registered with UMIN (UMIN000048174).

Results

A total of 18 patients were enrolled and eight were assigned to the green-tea-drinking and 10 to the water-drinking group. Patient characteristics were similar in both groups. The time to first defecation was 42.0 ± 21.8 hours in the green-tea-drinking and 49.4 ± 42.2 hours in the water-drinking group (p=0.79). There were also no significant differences in postoperative hospital stay (p=0.28) or mean daily fluid intake (p=0.07). In the green-tea-drinking group, one patient developed postoperative ileus, and another experienced pseudogout, showing no significant difference compared with the water-drinking group (p=0.09).

Conclusions

In this pilot single-center stratified randomized controlled trial, drinking either green tea or water after laparoscopic or open colon cancer surgery was safe and made no difference to the primary or secondary outcomes.

## Introduction

Colorectal cancer (CC) is the third most common cancer worldwide, ranking as the third most prevalent in men and the second in women, and it has the second highest mortality rate among cancers [1]. In 2020, it was estimated that over 1.9 million new cases of CC occurred, leading to approximately 935,000 deaths [1]. Multiple factors contribute to the development of CC, but surgical resection remains the cornerstone for improving survival outcomes in patients with advanced CC [2]. However, colorectal surgery is considered a high-risk procedure, with reported morbidity and mortality rates after radical colectomy at approximately 10% and 5%, respectively [3]. Notably, postoperative ileus (POI) occurs in 10-15% of colorectal surgery cases, substantially extending the length of hospital stay and exacerbating the economic burden on public health systems [4-7]. Devising ways to quicken patient recovery, decrease the incidence of postoperative complications, and reduce patient distress remains a crucial area of ongoing medical research [8].

Since the introduction of Enhanced Recovery Protocols, multimodal strategies have been employed to improve gastrointestinal function (GIF) after surgery [8]. Our previous systematic review and meta-analysis indicated that postoperative coffee consumption is effective in reducing POI following abdominal surgeries such as colorectal operations, cesarean sections, and gynecological surgeries [9]. However, what kind of foods and liquids should be used as standards for early intake continues to be debated. Green tea, commonly consumed in Japan, China, and other East Asian countries, is rich in catechins, various amino acids, and caffeine, possessing antioxidant, anti-inflammatory, hypocholesterolemic, anti-atherosclerotic, and antimicrobial pharmacological properties [10]. A previous study has reported that drinking Chinese green tea after subtotal gastrectomy is safe and can promote postoperative recovery of GIF [11]. However, it is unclear whether drinking green tea can promote the recovery of postoperative GIF after colon cancer surgery. Since the gastrointestinal tract often directly interacts with high concentrations of green tea solutions and their components, regardless of absorption, retention, or recirculation, drinking green tea could potentially have a significant impact [11-13].

Therefore, we hypothesized that green tea consumption could improve short-term outcomes in patients who have undergone a colectomy for colon cancer. This pilot trial aimed to assess the impact and safety of drinking green tea on short-term outcomes following colon cancer surgery.

## Materials and methods

Study population

This pilot single-center prospective randomized controlled trial, conducted at the Tochigi Medical Center Shimotsuga, Tochigi, Japan, included 18 patients who underwent colon cancer resection without preoperative neoadjuvant therapy between April 2023 and March 2024. Due to the absence of prior studies investigating postoperative green tea consumption for colon cancer, a formal sample size calculation could not be performed. Consequently, this study was designed as a pilot randomized controlled trial, with the number of cases determined by the 1-year period approved by the ethics committee. The protocol was registered with UMIN (UMIN000048174).

Inclusion criteria were as follows: 1. Men and women aged 18 years or older at the time of colon surgery; 2. Patients diagnosed histologically with colon cancer including cancers of the cecum, ascending colon, transverse colon, descending colon, and sigmoid colon.

The following patients were excluded: 1. Patients unable or unwilling to provide informed consent or adhere to the study protocol; 2. Patients exhibiting issues with mental state or language abilities; 3. Patients participating in another interventional trial; 4. Patients requiring emergency surgery; 5. Patients requiring multiple organ resections during surgery; 6. Patients requiring the creation or closure of an intestinal fistula during surgery; 7. Patients requiring the construction of a stoma; 8. Patients displaying complete obstruction; 9. Patients with known hypersensitivity or allergy to caffeine or green tea; 10. Patients with a physical status score of IV or V according to the American Society of Anesthesiologists (ASA); 11. Patients with alcohol dependence or substance abuse; 12. Patients with regular consumption of more than 800 mg of caffeine per day (equivalent to 8-10 cups of coffee); 13. Patients with an extensive history of abdominal surgery excluding appendectomy, cholecystectomy, hernia repair, and cesarean section; 14. Patients with heart failure of grade III or higher according to the New York Heart Association; 15. Patients using opioid analgesics or >5 mg/day of steroids in the 7 days prior to surgery; 16. Patients with long-term use of glucocorticoids or stimulant laxatives; 17. Patients using antidepressants; 18. Patients with severe obesity (body mass index [BMI] > 32) or severe malnutrition (BMI < 15); 19. Patients displaying a hypertensive emergency (blood pressure above 180/120 mmHg); 20. Patients with a potential for pregnancy, breastfeeding, or childbirth; 21. Patients with liver failure and/or cirrhosis (Model for End-Stage Liver Disease score >15); and 22. Patients using drugs that are substrates or inhibitors of the CYP1A2 enzyme (such as fluoroquinolone antibiotics).

Randomization and masking

Random sequence generation was conducted using a computer-generated random number table to ensure random allocation of participants to the intervention or control group, stratified by age (75 years old or younger) and with or without sigmoid colon resection. Allocation concealment was achieved using sequentially numbered, opaque, sealed envelopes to prevent selection bias. Blinding of participants and attending clinicians was not feasible due to the nature of the intervention. However, outcome assessments were conducted by nurses who were blind to the group assignments, ensuring objective evaluation of the study endpoints.

Intervention and control

The intervention was defined as the consumption of 600 mL of Suntory Green Tea Lemon Intense Flavor®️ (Suntory Beverage & Food Limited, Tokyo, Japan) daily, starting from the first postoperative day. The control treatment was defined as consuming 600 mL of water per day (divided into three 200 mL servings) starting from the first postoperative day. The volume consumed was verified by measuring the amount remaining in the container. The intervention period extended from the first postoperative day until the first bowel movement occurred. Additional hydration was permitted. Postoperative oral intake followed the Enhanced Recovery Protocols, transitioning from liquid to solid foods based on food tolerance, with a focus on safety. If no bowel movement occurred within the first four days post surgery, the use of laxatives or enemas was allowed.

Outcomes

The primary outcome was time to first defecation (hours), defined as time (hours) from the end of surgery to first defecation. The secondary outcomes were the length of hospital stay, POI within 30 days postoperatively, postoperative complications based on Clavien-Dindo classification of ≥Ⅱ within 30 days postoperatively, the mean volume of fluid intake per day (mL), and the incidence of all adverse events.

Surgery

All surgeries were conducted by the same surgical team. Current evidence suggests that there is no significant difference in postoperative complications and short-term or long-term outcomes between laparoscopic colectomy and open surgery for colon cancer. The choice between laparoscopic or open surgery was made by the patients and their families at the time of obtaining surgical consent, based on the principle of therapeutic intent. All patients had undergone a standard colectomy, which may have involved either mechanical or hand-sewn anastomosis. The surgical techniques and lymph node dissection were performed according to the Japanese Guidelines for the Treatment of Colorectal Cancer, 2019 edition [2]. This ensured that all procedures adhered to the highest standards of current medical practice and guidelines, promoting consistency and reliability in surgical outcomes.

Statistical analysis

Data were analyzed using intention-to-treat principles. Continuous variables were summarized using means and standard deviations (SD), while categorical variables were summarized using frequencies and percentages. Comparisons between groups were conducted using Pearson’s chi-square test or the Mann-Whitney U-test, with a significance level set at p<0.05. All results were analyzed using the intention-to-treat analysis. Sensitivity analysis was performed using a per protocol analysis excluding patients with pathological stage (pStage) IV. All statistical analyses were performed using STATA SE16 software (version 16.1, Stata Corporation, College Station, TX, USA).

Ethical considerations

This study was approved by the Institutional Review Board of Tochigi Medical Center Shimotsuga, Tochigi, Japan (Approval Number: 218). Study procedures were carried out in accordance with the Declaration of Helsinki. All participants provided written informed consent before enrollment.

## Results

**Figure 1:**
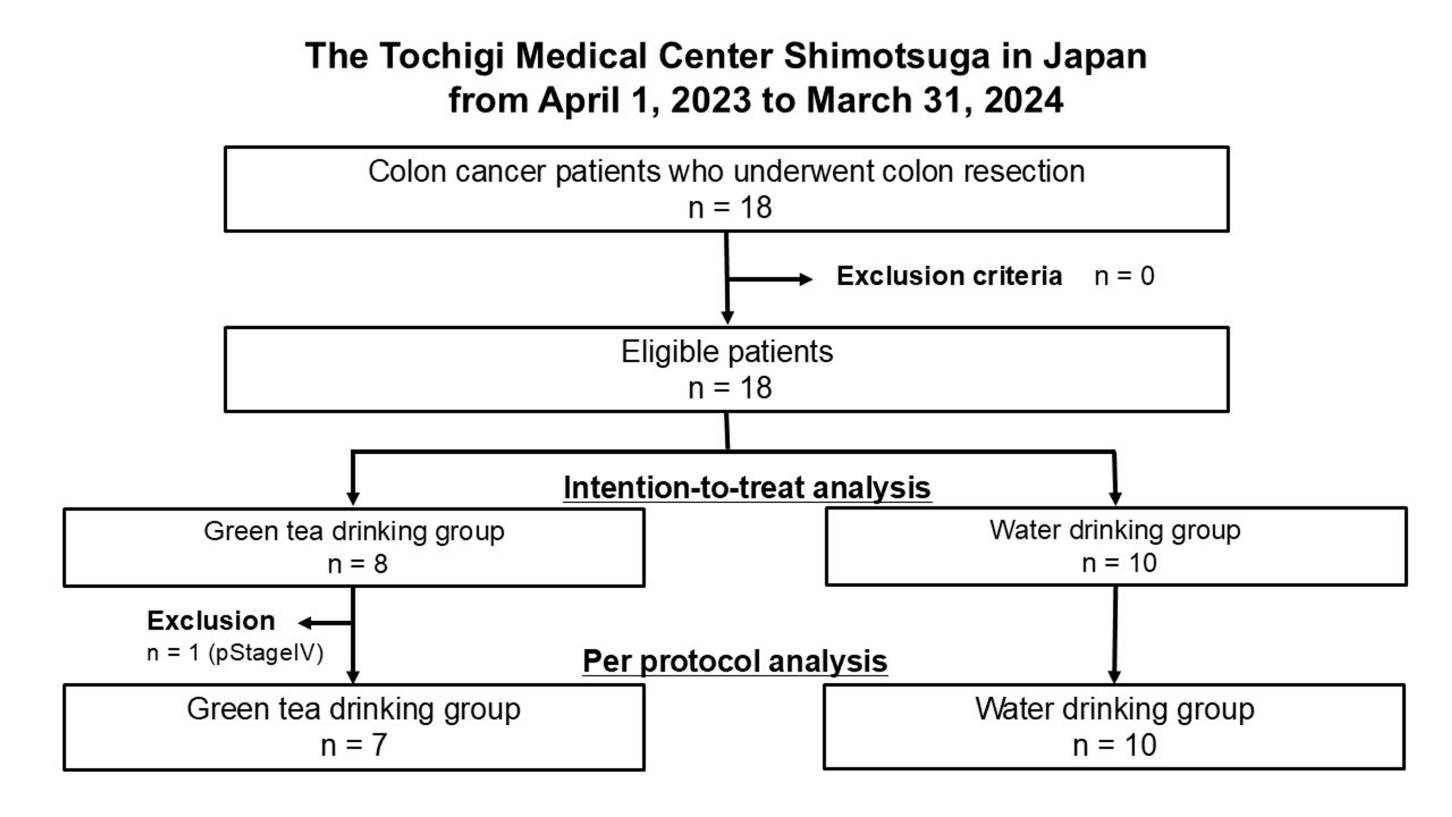
Flowchart of enrolled patients with colon cancer who underwent colon surgery between 2023-2024 at the Tochigi Medical Center Shimotsuga

A total of 18 patients were randomly assigned to the two groups (Figure 1). The comparison between the green-tea-drinking (N=8) and water-drinking (N=10) groups revealed no statistically significant differences across clinicopathological factors, including age, sex, BMI, ASA score, smoking and drinking status, serum carcinoembryonic antigen and carbohydrate antigen 19-9 levels, surgical interventions (sigmoid colon resection and laparoscopy), and pathological stage (Table 1).

**Table 1:** Clinicopathological factors of patients between green tea and water in intention-to-treat analysis. ASA-PS, American Society of Anesthesiologists physical status; BMI, body mass index; mCCI, modified Charlson comorbidity index; SD, standard deviation; CEA, Carcinoembryonic Antigen; CA19-9, Carbohydrate Antigen 19-9; pStage, pathological stage. * indicates p < 0.05, Mann-Whitney U test or Chi-square test.

Variables	Green tea N=8	Water N=10	p-Value*
Age (y), mean (SD)	74.6 (6.1)	68.7 (10.2)	0.17
Male, n (%)	4 (50)	4 (40)	0.67
BMI (kg×m^-2^), mean (SD)	20.6 (2.8)	22.5 (3.7)	0.25
mCCI	5.9 (0.8)	4.7 (1.4)	0.06
ASA-PS score	1.9 (0.6)	1.6 (0.5)	0.33
Current smoker, n (%)	3 (38)	3 (30)	0.18
Current drinker, n (%)	4 (50)	2 (20)	0.18
CEA (ng/mL)	3.0 (1.7)	5.5 (7.5)	0.36
CA19-9 (U/mL)	0.8 (0.2)	0.7 (0.1)	0.12
Sigmoid colon resection, n (%)	2 (25)	2 (20)	0.06
Laparoscopy, n (%)	3 (38)	7 (70)	0.17
pStage (I/II/III/ IV)	1/3/3/1	2/4/4/0	0.70

Primary outcome

The primary outcome, time to first defecation post-surgery, was similar between the two groups. The mean time to first defecation was 42.0 hours (SD 21.8) in the green tea group and 49.4 hours (SD 42.2) in the water group, with no statistically significant difference (p=0.79) (Table 2).

**Table 2:** Outcomes in intention-to-treat analysis SD, standard deviation. * indicates p < 0.05, Mann-Whitney U test or Chi-square test.

Variables	Green tea N=8	Water N=10	p-Value*
First defecation (hours), mean (SD)	42.0 (21.8)	49.4 (42.2)	0.79
Length of hospital stay (days), mean (SD)	10.1 (2.3)	8.9 (1.1)	0.28
Postoperative ileus, n (%)	1 (13)	0 (0)	0.25
Postoperative complications, n (%)	1 (13)	0 (0)	0.25
Mean amount of drinking per day (mL), mean (SD)	386 (218)	545 (121)	0.07
All adverse events, n (%)	2 (25)	0 (0)	0.09

Secondary outcomes

Secondary outcomes also showed no statistically significant differences between the groups. The mean length of hospital stay was 10.1 days (SD 2.3) in the green tea group compared to 8.9 days (SD 1.1) in the water group (p=0.28). One patient (13%) in the green tea group experienced POI, with no such events reported in the water group (p=0.25 for both comparisons). The mean amount of fluid intake per day was lower in the green tea group (386 mL, SD 218) compared to the water group (545 mL, SD 121), with the difference approaching statistical significance (p=0.07). Overall, adverse events, including POI and pseudogout requiring analgesics, occurred in 25% of patients in the green tea group, with no adverse events reported in the water group (p=0.09).

Per protocol analysis

The sensitivity evaluation using per protocol analysis, which excluded patients with pStage IV, yielded results consistent with the primary analysis. There were no significant differences between the green-tea-drinking and water-drinking groups across all measured outcomes (Table 3).

**Table 3:** Outcomes in per protocol analysis SD, standard deviation. * indicates p < 0.05, Mann-Whitney U test or Chi-square test.

Variables	Green tea N=7	Water N=10	p-Value*
First defecation (hours), mean (SD)	45.0 (22.7)	49.4 (42.2)	0.94
Length of hospital stay (days), mean (SD)	10.5 (3.0)	8.9 (1.1)	0.45
Postoperative ileus, n (%)	0 (0)	0 (0)	1.00
Postoperative complications, n (%)	1 (14)	0 (0)	0.12
Mean amount of drinking per day (mL), mean (SD)	512 (103)	545 (121)	0.41
All adverse events, n (%)	1 (14)	0 (0)	0.12

## Discussion

In this pilot randomized controlled trial involving 18 patients, we found no statistically significant differences between the green-tea-drinking and water-drinking groups in terms of the primary outcome, time to first defecation post-surgery. The mean time to first defecation was similar between the groups, suggesting that green tea consumption did not impact this critical postoperative recovery marker. Secondary outcomes, including the length of hospital stay and the occurrence of postoperative complications, such as POI, were also comparable between the two groups. Although the green-tea-drinking group had a lower mean daily fluid intake and a higher incidence of adverse events-including POI and pseudogout requiring analgesics - these differences were not statistically significant. These findings suggest that green tea may not significantly influence postoperative recovery outcomes compared to water, but further research with larger sample sizes is warranted to validate these observations.

In contrast to previous studies, such as our systematic review showing that coffee consumption significantly reduces POI across various abdominal surgeries [9], and a randomized controlled trial indicating that green tea improves gastrointestinal recovery following subtotal gastrectomy [11], the present pilot trial did not demonstrate a similar benefit of green tea after colon cancer surgery. The lack of a significant effect in our study may be due to differences in surgical procedures, with colectomy presenting distinct postoperative challenges compared to gastrectomy [14,15]. Additionally, the small sample size in our study may have limited the detection of subtle effects of green tea on postoperative outcomes. Further research is needed to clarify the role of green tea in postoperative recovery, particularly in the context of colorectal surgery.

Although the exact mechanisms underlying the differences in time to first flatus between gastrectomy and colectomy are not fully understood, several factors may contribute. After gastrectomy, the residual stomach and small intestine may recover motility more quickly due to the stimulatory effects of caffeine and catechins in green tea, which enhance vagal nerve activity and reduce the inhibitory effects of nitric oxide on gastrointestinal motility [16-18]. This could lead to a shorter time to the first flatus. In contrast, colectomy involves the colon, which has a slower, reflex-dependent motility [19]. The disruption of local nerve reflexes and the dependence of the colon on the enteric nervous system may delay the recovery of bowel function, extending the time to the first flatus [20]. These observations suggest that the benefits of green tea and its components may vary depending on the specific surgical context.

Limitations

This pilot study has several limitations that should be considered. First, as a single-center study with a small sample size, the statistical power to detect differences in postoperative outcomes, including complications and gastrointestinal recovery, is limited. Additionally, while both green tea and water were provided at room temperature, the distinct taste and aroma of green tea could not be blinded, potentially introducing a social desirability bias where patients may report outcomes they perceive as favorable. Further, various perioperative factors, such as individual differences in surgical technique and patient comorbidities, may influence GIF and potentially dilute the observed effects of green tea. Although we aimed to control confounding variables, the underlying mechanisms by which green tea may enhance gastrointestinal recovery remain elusive and warrant further investigation. Finally, our study excluded patients with significant comorbidities, which limits the generalizability of our findings to the broader population undergoing colorectal surgery. Future studies with larger, more diverse populations and multicenter designs are needed to validate these findings and explore the potential benefits of green tea in postoperative recovery.

## Conclusions

This pilot randomized controlled trial found no statistically significant difference in the primary outcome, time to first defecation post-surgery, between the green-tea-drinking and water-drinking groups. The mean time to first defecation was similar between both groups, indicating that green tea consumption did not significantly impact this crucial marker of postoperative recovery following colectomy for colon cancer. Our current findings do not support the hypothesis that green tea can improve short-term gastrointestinal recovery outcomes. However, due to the limitations of this study, including the small sample size and single-center design, further research is warranted. Larger studies are needed to confirm these findings and explore whether different conditions or patient populations may reveal potential benefits of green tea in postoperative care. Despite the lack of significant results in this trial, the well-documented health benefits of green tea suggest that its role in enhancing postoperative recovery remains a promising area for future research.
